# Complex transsphincteric ano-rectal fistula with circumferential perirectal abscess and periprostatic extension: the added value of contrast-enhanced pelvic MRI

**DOI:** 10.1093/bjrcr/uaag018

**Published:** 2026-04-28

**Authors:** Chi Phong Nguyen, Quang Huy Huynh

**Affiliations:** Radiology Department, Binh Dan Hospital, Ho Chi Minh City, 700000, Viet Nam; Radiology Department, Binh Dan Hospital, Ho Chi Minh City, 700000, Viet Nam; Radiology Department, Pham Ngoc Thach University of Medicine, Ho Chi Minh City, 700000, Viet Nam; Radiology Department, University of Health Sciences-Viet Nam National University, Ho Chi Minh City, 700000, Viet Nam

**Keywords:** ano-rectal fistula, perirectal abscess, pelvic MRI, transsphincteric fistula, surgical planning

## Abstract

Complex perianal fistulas can harbor extensive secondary extensions and pelvic sepsis despite minimal systemic symptoms and normal inflammatory markers, creating a high risk of incomplete surgery when anatomy is underestimated. We report a case of a complex transsphincteric fistula (Parks classification) with suspected supralevator involvement in which preoperative MRI provided decisive anatomic mapping for operative planning. Multiplanar T2-weighted imaging delineated the primary tract, its relationship to the sphincter complex and levator plate, and extension into the ischioanal/ischiorectal fossae, while also demonstrating features consistent with secondary tracts and occult abscesses—findings that commonly explain postoperative persistence or recurrence if missed. Notably, gadolinium-enhanced fat-suppressed T1-weighted sequences increased conspicuity of subtle distal rectal communications that were poorly visualized on T2-weighted images, supporting the utility of contrast in selected scenarios to distinguish enhancing active tracts/abscess walls from surrounding inflammatory edema or fibrosis. Given concomitant distal rectal ulceration on endoscopy, an inflammatory bowel disease work-up was considered to exclude fistulizing Crohn’s disease, for which careful staging of perianal sepsis and combined medical–surgical management are recommended. This case highlights the value of MRI (including contrast-enhanced protocols when indicated) as a preoperative adjunct to examination under anesthesia, enabling tailored, sphincter-preserving strategies and comprehensive sepsis control in complex fistulizing disease.

## Introduction

Ano-rectal fistula is commonly the chronic sequela of cryptoglandular infection and may be complicated by secondary tracts and abscesses, which are major drivers of persistent sepsis and recurrence after surgery.^1^ Surgical success depends on accurate identification of the internal opening, the relationship of the primary tract to the sphincter complex, and the detection of any secondary extensions or supralevator/pelvic spread.^2^

MRI has become the preferred preoperative imaging technique for complex perianal fistulas because it provides multiplanar mapping of fistula anatomy, depicts the levator plate and pelvic compartments, and identifies occult abscesses that may be missed on examination under anesthesia (EUA) alone.^3^ Contrast-enhanced sequences may further increase confidence in differentiating active granulation tissue and enhancing abscess walls from adjacent edema or fibrosis, and may reveal subtle communicating tracts not conspicuous on T2-weighted images.^4^ Evidence suggests that MRI-informed surgery reduces recurrence in recurrent fistula-in-ano compared with surgery not guided by imaging.^5^ Current clinical practice guidelines recommend drainage of abscess/sepsis and sphincter-preserving strategies (eg, seton) for complex fistulas, with imaging playing an important role in preoperative planning.^6^

## Case description

A 40-year-old man presented with persistent anal discharge without fever. Laboratory evaluation was unremarkable (WBC 6.66 K/µL; neutrophils 52.5%).

Pelvic MRI was performed first for anatomical assessment of the fistulous disease. The examination included multiplanar T1- and T2-weighted sequences, fat-suppressed T2-weighted images, and gadolinium-enhanced fat-suppressed T1-weighted sequences.

**Figure 1:**
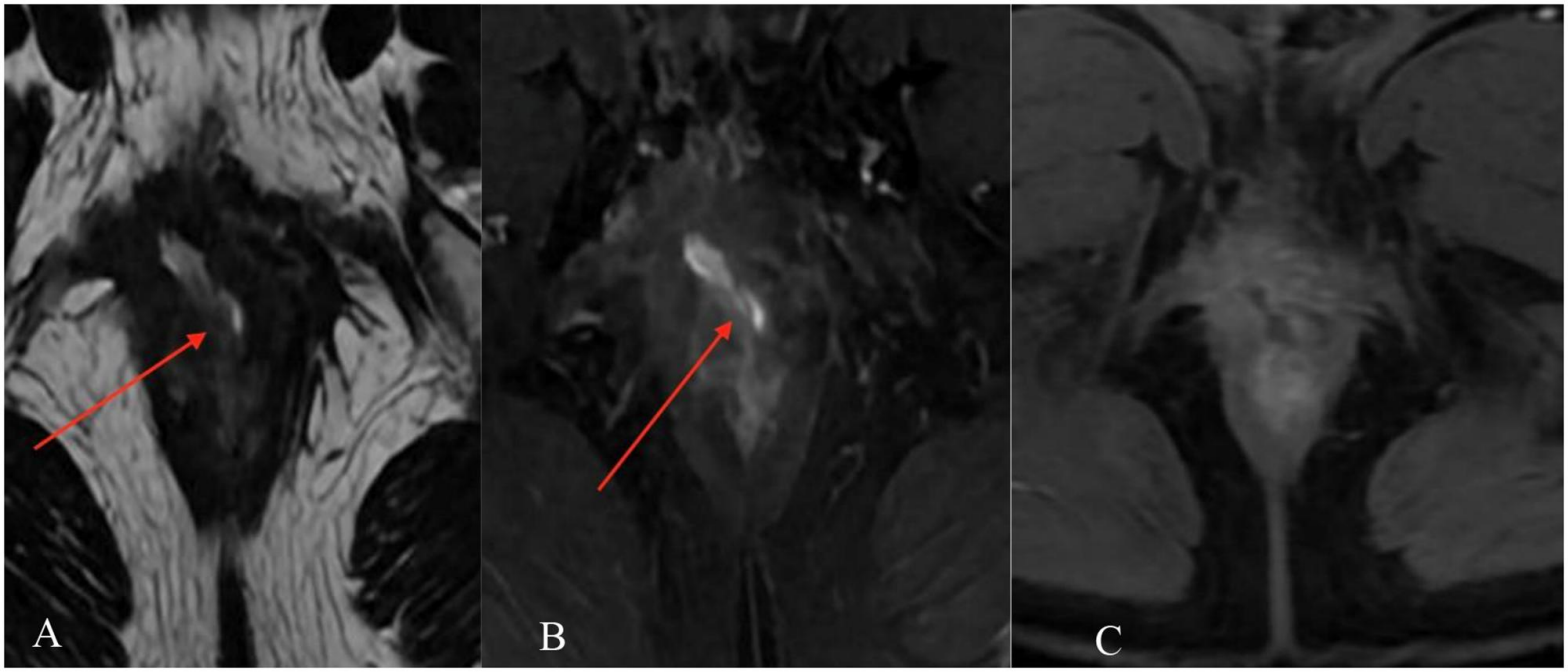
Primary transsphincteric fistula and internal opening on pelvic MRI. (A) Axial T2-weighted image shows the internal opening at approximately the 11 o’clock position (arrow). (B) Coronal fat-suppressed T2-weighted image demonstrates the fistulous tract extending anterolaterally to the right (arrow). (C) Coronal T1-weighted image provides an anatomic overview of the anorectal region and adjacent pelvic structures.

**Figure 2:**
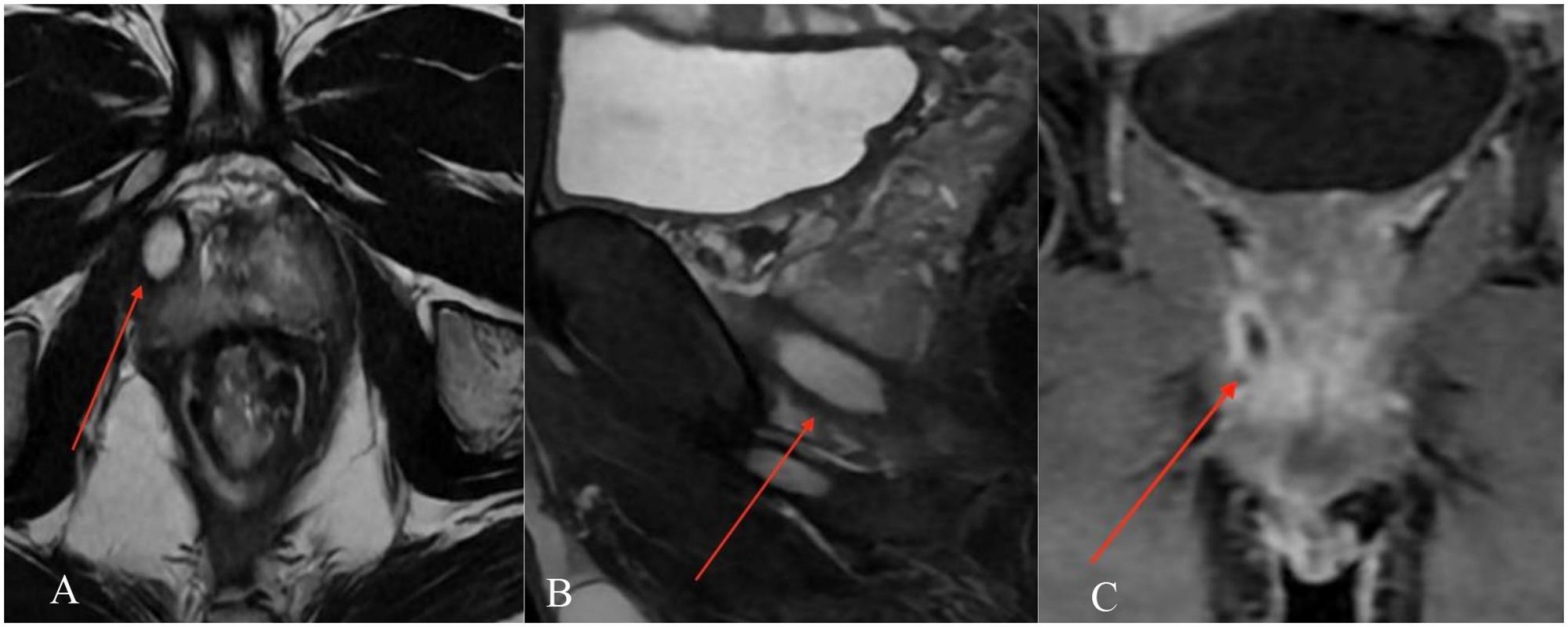
Additional right-sided extension of the fistulous disease. (A) Axial T2-weighted image shows a rounded hyperintense right perianal/perirectal collection or tract component (arrow). (B) Sagittal T2-weighted image demonstrates cranial extension of the abnormal tract/collection along the right pelvic sidewall toward the inferior periprostatic region (arrow). (C) Coronal post-contrast fat-suppressed T1-weighted image shows enhancement around the right-sided tract/collection, supporting active inflammatory change (arrow).

**Figure 3:**
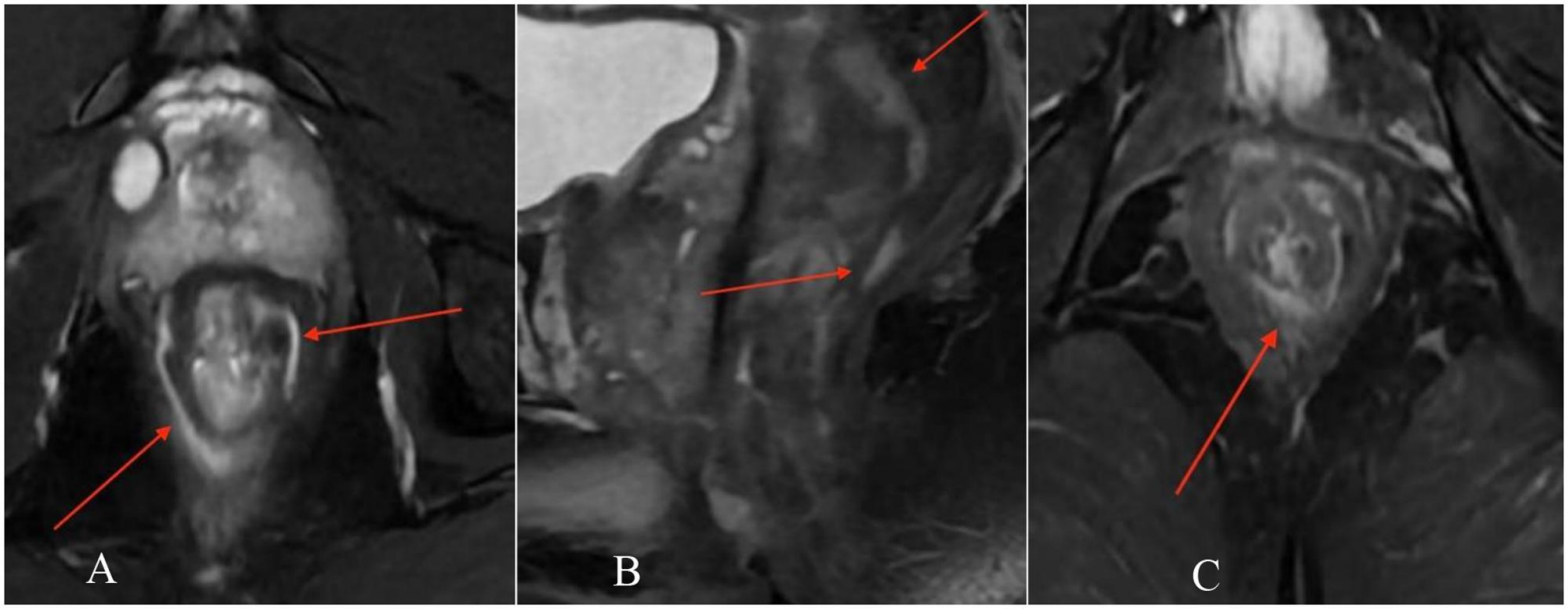
Circumferential perirectal inflammatory collection with extensive pelvic involvement. (A) Axial T2-weighted image demonstrates circumferential hyperintense perirectal collection surrounding the rectum, with associated mural thickening and inflammatory change (arrows). (B) Sagittal T2-weighted image shows longitudinal extent of the perirectal inflammatory process and associated mesorectal/perirectal edema (arrows). (C) Coronal fat-suppressed T2-weighted image depicts the circumferential perirectal collection and surrounding inflammatory change in the lower pelvis (arrow).

**Figure 4:**
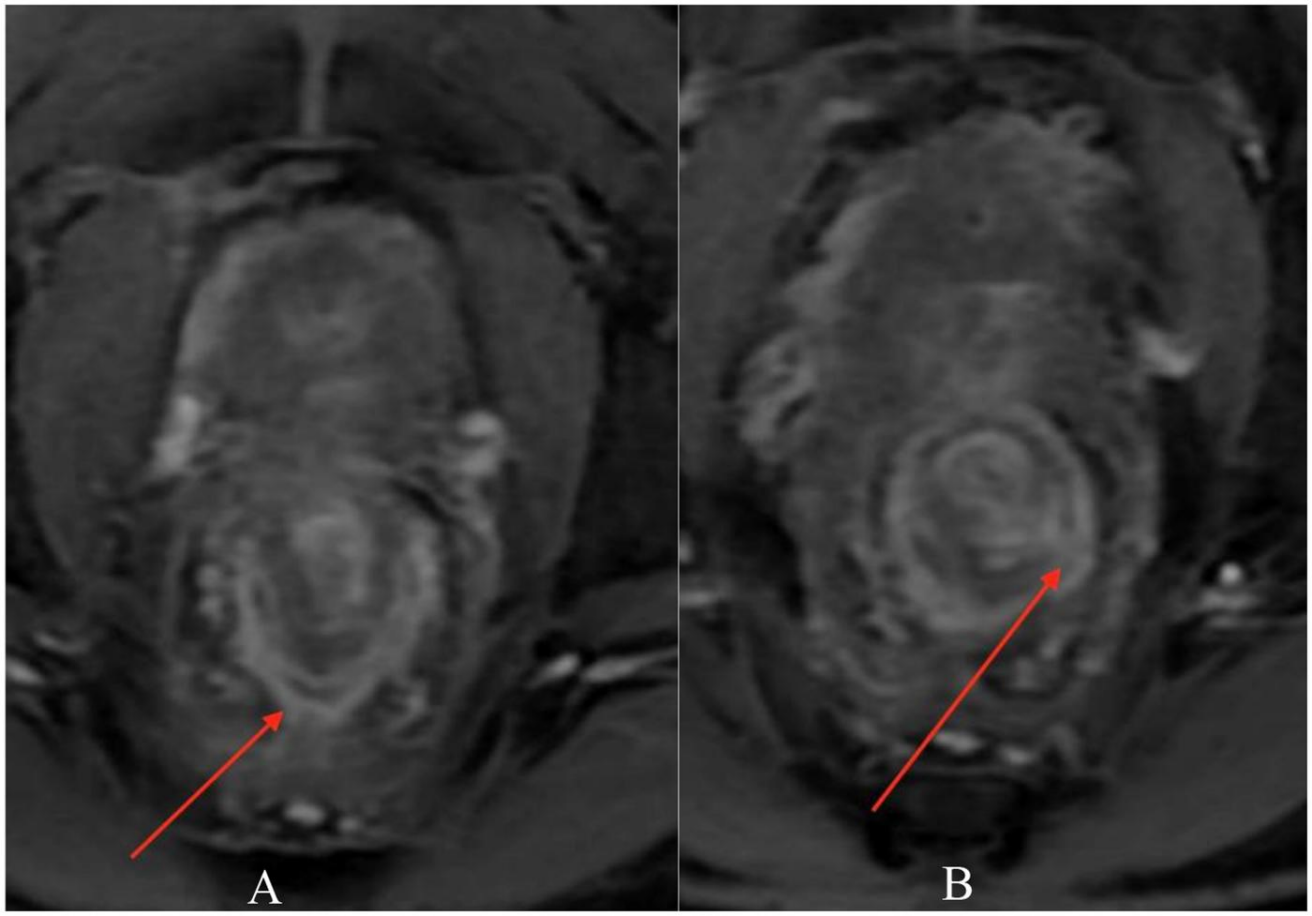
Post-contrast depiction of subtle rectal communication and circumferential perirectal enhancement. (A) Coronal post-contrast fat-suppressed T1-weighted image shows peripheral enhancement around the circumferential perirectal collection, compatible with inflammatory abscess wall/granulation tissue (arrow). (B) Coronal post-contrast fat-suppressed T1-weighted image demonstrates a subtle suspected communication between the rectal lumen and the perirectal collection that is better appreciated after contrast administration (arrow).

MRI demonstrated a complex transsphincteric ano-rectal fistula with an internal opening at approximately the 11 o’clock position, about 12 mm from the anal verge, extending anterolaterally to the right (Figure 1). An additional infected extension tracked cranially along the right pelvic sidewall toward the inferior right periprostatic region (Figure 2). MRI also showed a circumferential perirectal inflammatory collection extending from the upper rectum to the intersphincteric space, with associated mesorectal fat stranding and perineal soft-tissue edema (Figure 3). On post-contrast images, there was peripheral enhancement around the tract/collection and subtle suspected communication between the rectal lumen and the perirectal collection that was less conspicuous on T2-weighted sequences (Figure 4).

Based on MRI morphology, the fistula was classified as transsphincteric according to the Parks classification,^7^ and was consistent with a high-grade fistula on the St James’s University Hospital MRI classification^8^ because of the associated abscess/secondary extension.

Subsequently, on the day of the planned intervention, endoscopy demonstrated ulcerative inflammation of the distal rectum and raised suspicion of a healing complex rectal fistula.

The patient initially received medical treatment. Forty-five days later, he underwent surgical debridement/excision of fibrotic fistula tissue with seton placement. The subtle rectal communications suggested on post-contrast MRI were not definitively confirmed intraoperatively or endoscopically.

## Discussion

This case illustrates a key clinical challenge: extensive fistulizing disease and pelvic sepsis may occur despite minimal systemic symptoms and normal inflammatory markers, making, preoperative anatomic mapping essential. The fistula was transsphincteric (Parks classification),^7^ but MRI revealed a disease burden beyond what would be expected from a straightforward transsphincteric tract—namely a circumferential perirectal abscess extending to the intersphincteric space and an atypical cranial extension toward the periprostatic region along the pelvic sidewall. Based on the transsphincteric tract with associated abscess/secondary extension, this case is also consistent with a high-grade fistula on the St James’s University Hospital MRI classification.^8^

MRI is well established as the most informative modality for complex perianal fistulas because it demonstrates the sphincter complex, the levator plate, and the ischioanal/ischiorectal fossae, while also detecting secondary tracts and occult abscesses that frequently explain postoperative persistence or recurrence.^9^ The St James’s MRI-based grading system emphasizes increasing complexity with secondary extensions/abscesses and supralevator spread, precisely the elements that determine operative strategy.^8^

In our patient, gadolinium-enhanced images suggested additional subtle suspected rectal communications that were not well visualized on T2-weighted sequences; however, these were not definitively confirmed at surgery or endoscopy. This finding is consistent with prior work supporting the utility of contrast-enhanced MRI protocols—particularly in differentiating active tracts/abscess walls from surrounding inflammatory edema or fibrosis and in improving tract conspicuity in selected scenarios.^4^ Importantly, MRI has been shown to impact management and outcomes: in recurrent fistula-in-ano, surgery guided by MRI findings is associated with substantially fewer further recurrences compared with surgery that does not act on imaging.^3^ A meta-analysis has also demonstrated strong diagnostic performance for MRI in perianal fistula assessment, supporting its role as a preoperative adjunct alongside EUA and other modalities.^10^ In practice, because the full extent of disease and the presence of subtle secondary communications are often not known before imaging, this case supports inclusion of contrast-enhanced sequences in pelvic MRI protocols for complex fistulizing disease.

From a therapeutic standpoint, contemporary guidelines recommend prompt control of sepsis (abscess drainage) and sphincter-preserving approaches (eg, seton) for complex fistulas to minimize incontinence risk, with definitive procedures individualized based on anatomy and underlying etiology.^1^ Because endoscopy showed distal rectal ulceration, an inflammatory bowel disease (IBD) work-up should be considered, as perianal fistulizing Crohn’s disease requires combined medical–surgical management and careful staging of perianal sepsis.^11^ In our patient, MRI findings helped define the extent of disease and supported operative planning with debridement and seton placement. Even when cryptoglandular disease is suspected, the presence of extensive pelvic extension on MRI should prompt clinicians to search for contributing factors (IBD, immunosuppression, prior interventions) and to ensure comprehensive drainage and follow-up imaging when clinically indicated.

Overall, this case supports routine use of high-quality pelvic MRI (including contrast-enhanced sequences when helpful) to map fistula anatomy and sepsis extent, enabling tailored surgical planning and potentially reducing persistent infection and recurrence.

## Conclusion

Contrast-enhanced pelvic MRI was pivotal in this case by clearly delineating a complex transsphincteric fistula with a well-localized internal opening, demonstrating an extensive circumferential perirectal abscess extending to the intersphincteric space, and revealing an atypical cranial extension along the right pelvic sidewall to the periprostatic region with subtle additional rectal communications that were not apparent on T2-weighted images. Comprehensive MRI mapping enabled appropriate surgical planning with sepsis control and a sphincter-preserving approach using seton placement, thereby helping to reduce the risk of persistent infection and recurrence.

Learning points

- Contrast-enhanced pelvic MRI can improve delineation of complex ano-rectal fistula anatomy, including active tracts, abscess walls, and subtle secondary communications.
- Pelvic MRI can detect atypical extensions, including pelvic sidewall or periprostatic spread, even when systemic symptoms and inflammatory markers are minimal.
- Detailed preoperative MRI mapping helps guide sepsis control and sphincter-preserving surgical planning in complex fistulizing disease.
